# Mini-Incision Versus Laparoscopic Cholecystectomy

**DOI:** 10.1155/1997/27210

**Published:** 1997

**Authors:** Charles J. Yeo

**Affiliations:** Johns Hopkins University School of Medicine Blalock 606 600 N Wolfe Street Baltimore MD 21287- 4606 USA

## Abstract

*Background*: We report a prospective randomised comparison between laparoscopic and small-incision cholecystectomy in 200 patients which was designed to eliminate bias for or against either technique.

*Methods*: Patients were randomised in the operating theatre and anaesthetic technique and pain-control methods were standardised. Four experienced surgeons did both types of procedure. Identical wound dressings were applied in both groups so that carers could be kept blind to the type of operation.

*Findings*: There was no significant difference between the groups for age, sex, body mass index, and American Society of Anaesthesiologists grade. Laparoscopic cholecystectomy took significantly longer than small-incision cholecystectomy (median 65 [range 27-140] min *vs* 40 [18-142] min, *p*<0.001). The operating time included operative cholangiography which was attempted in all patients. We found no significant difference between the groups for hospital stay (postoperative nights in hospital, median 3.0 [1-17] nights for laparoscopic *vs* 3.0 [1-14] nights for small-incision, *p*=0.74), time back to work for employed persons (median 5.0 weeks *vs* 4.0 weeks; *p*=0.39), and time to full activity (median 3.0 weeks *vs* 3.0 weeks; *p*=0.15).

*Interpretation*: Laparoscopic cholecystectomy takes longer to do than small-incision cholecystectomy and does not have any significant advantages in terms of hostital stay or 13 ostoperative recovery.


[3] Abelo, M. D., Armitage, J. O., Lichter, A. S. and Niederhuber, J. E. (eds) (1995). *Clinical Oncology*. Churchill Livingston Inc.
[4] Ouchi, K., Suzuki, M. and Saijo, S. *et al.* (1993). Do recent advances in diagnosis and operative management improve the outcome of gallbladder? *Surgery*, **113**(3), 324–329.
[5] Wanebo, H. J., Castle, W. N. and Fechner, R. E. (1982). Is carcinoma of the gallbladder a curable lesion? *Ann. Surg.*, **195**, 624–631.

M Margaret Kemeny, MD
North Shore University Hospital
Department of Surgery
Cornell University Medical College
300 Community Drive
Manhasset
New York 11030
United States of America

### Mini-Incision Versus Laparoscopic Cholecystectomy

### ABSTRACT

*Majeed, A. W., Troy, G., Nicholl, J. P., Smythe, A., Reed, M. W. R., Stoddard, C. J., Peacock, J. and Johnson A. G. (1996) Randomised, prospective, single-blind comparison of laparoscopic versus small-incision cholecystectomy. The Lancet; 347,* 989–994.

***Background:*** **We report a prospective randomised comparison between laparoscopic and small-incision cholecystectomy in 200 patients which was designed to eliminate bias for or against either technique.**
***Methods:*** **Patients were randomised in the operating theatre and anaesthetic technique and pain-control methods were standardised. Four experienced surgeons did both types of procedure. Identical wound dressings were applied in both groups so that carers could be kept blind to the type of operation.**
***Findings:*** **There was no significant difference between the groups for age, sex, body mass index, and American Society of Anaesthesiologists grade. Laparoscopic cholecystectomy took significantly longer than small-incision cholecystectomy (median 65 [range 27–140] min *vs* 40 [18–142] min, $p<0.001$). The operating time included operative cholangiography which was attempted in all patients. We found no significant difference between the groups for hospital stay (postoperative nights in hospital, median 3.0 [1–17] nights for laparoscopic *vs* 3.0 [1–14] nights for small-incision, $p=0.74$), time back to work for employed persons (median 5.0 weeks *vs* 4.0 weeks; $p=0.39$), and time to full activity (median 3.0 weeks *vs* 3.0 weeks; $p=0.15$).**
***Interpretation:*** **Laparoscopic cholecystectomy takes longer to do than small-incision cholecystectomy and does not have any significant advantages in terms of hospital stay or postoperative recovery.**

*Keywords:* Laparoscopic cholecystectomy, mini-incision cholecystectomy

### PAPER DISCUSSION

Laparoscopic cholecystectomy serves as the prototype success story for the introduction of minimally invasive surgery into the mainstream practice of surgery world wide. This study by Majeed *et al.* recruited 200 patients over a three and one-half year period with symptomatic gallstones. Patients were randomized intra-operatively after the induction of anesthesia to undergo either standard laparoscopic cholecystectomy with an attempt at routine operative cholangiography, or "small-incision" cholecystectomy. The small incision used in this study was a high transverse sub-xiphoid incision, dividing the rectus muscle as needed, and dissecting the gallbladder from Calot's triangle toward the fundus with long instruments, avoiding the insertion of hands into the peritoneal cavity. An important aspect of this study is that the patients were treated post-operatively with a patient-controlled analgesic system delivering morphine, and that the patients were "told that they could get out of bed and go home as soon as they felt fit enough". Additionally, patients were "given no advice on how long they would expect to remain convalescent".

While initially 100 patients were randomized to the laparoscopic and "small-incision" arms respectively, 20 patients in the laparoscopic group were converted to open cholecystectomy and there was one bile duct injury in the laparoscopic cholecystectomy group. The median length of the incision in the "small-incision" group was 7 cm, ranging from 4 cm to 18 cm. Comparative data for all patients randomized to the laparoscopic versus "small-incision" groups (100 in each group) indicated that the operating time of 69 minutes was significantly longer in the laparoscopic group as compared to 45 minutes in the "small-incision" group. Additionally, the time to first feeding was 24.7 hours in the laparoscopic group versus 22.4 hours in the "small-incision" group, a difference which achieves statistical significance, but is of course meaningless. There were no significant differences in the hospital stay (approximately 3.5 nights in each group), time off work (approximately $4\frac{1}{2}$ to 5 weeks in each group), and the length of time required to return to full activity, (approximately 4 weeks in both groups).

The authors are to be congratulated for conducting this study which compares laparoscopic to "small-incision" cholecystectomy. While their data indicate that laparoscopic cholecystectomy requires additional operative time, their findings that it confers no benefit over "small-incision" cholecystectomy in terms of postoperative recovery, hospital stay and time back to work may not be applicable to patients in other settings, other countries, or patients operated upon outside of the British Health Service. For example, the policy of "self determination" used in this study regarding the patient's timing of ambulation and hospital discharge is not applicable to the vast majority of patients who undergo laparoscopic cholecystectomy in the United States. Laparoscopic cholecystectomy in the U.S. is typically attended by no more than a one night hospital stay, and in many settings is now performed as an outpatient procedure, with the patient being discharged on the same day as the procedure. Such an approach requires preoperative patient education, appropriate patient expectations, modifications in the anesthetic and analgesic regimens (to avoid the use of emesis-associated drugs), as well as the use of in- filtrative local anesthetics to reduce the pain associated with the laparoscopic incisions.

Several studies have now been reported comparing laparoscopic to open or "small-incision" cholecystectomy [1,2,3]. Additionally, laparoscopic and "small-incision" cholecystectomy have been compared using linear analogue pain scores, consumption of postoperative patient-controlled morphine, and pulmonary function [4]. A recent review of over 5,000 patients who underwent laparoscopic cholecystectomy in the U.S. Department of Defense Health Care System (at 89 military medical treatment facilities) indicated that the median length of stay for patients following laparoscopic cholecystectomy was one day [5], results identical to many of the recently published U.S. studies.

Majeed and coauthors are to be congratulated in performing a well designed study which compared laparoscopic to "small-incision" cholecystectomy within the structured confines of the trial design. While their data undoubtedly indicate that laparoscopic cholecystectomy utilizes more operative time than "small-incision" cholecystectomy, their conclusions that it has no advantages in terms of hospital stay or postoperative recovery require clarification. There is no doubt that in the proper setting, patients who are properly educated, appropriately motivated, and managed by surgeons and anesthesiologists with experience in outpatient procedures, can undergo laparoscopic cholecystectomy and commence oral intake within hours of their operative procedure, ambulate early, and be discharged to their homes for recovery in a familiar environment either on the day of their operation or one day later. The issue at hand now is to compare laparoscopic cholecystectomy to "small-incision" cholecystectomy in a properly designed trial, in such a setting.

### References

[1] Barkun, J. S., Barkun, A. N. and Sampalis, J. S. *et al.* and the McGill gallstone treatment group. (1992). Randomised controlled trial of laparoscopic versus mini cholecystectomy, *Lancet*, **340**, 1116-19.
[2] McMahon, A. J., Russel, I. T. and Baxter, J. N. *et al.* (1994). Laparoscopic versus minilaparotomy cholecystectomy: a randomised trial, *Lancet*, **343**, 135-38.
[3] McGinn, F. P., Miles, A. J. G., Uglow, M., Ozmen, M., Terzi, C. and Humby, M. (1995). Randomized trial of laparoscopic cholecystectomy and mini-cholecystectomy, *Br. J. Surg.*, **82**, 1374-77.
[4] McMahon, A. J., Russell, I. T. and Ramsay, G. *et al.* (1994). Laparoscopic and minilaparotomy cholecystectomy: A randomized trial comparing postoperative pain and pulmonary function, *Surgery*, **115**, 533-539.
[5] Wherry, D. C., Rob, C. G., Marohn, M. R. *et al.* (1994). An external audit of laparoscopic cholecystectomy performed in medical treatment facilities of the Department of Defense, *Ann. Surg.*, **220**, 626-634.

Charles J. Yeo, MD
Professor of Surgery
Johns Hopkins University
School of Medicine
Blalock 606
600 N Wolfe Street
Baltimore, MD 21287- 4606
United States of America

### Can Small Hepatocellular Carcinoma be Cured by Percutaneous Acetic Acid Injection Therapy?

### ABSTRACT

*Ohishi, K., Nomura, F., Ito, S. and Fujiwara, K. (1996) Prognosis of small hepatocellular carcinoma (less than 3cm) after percutaneous acetic acid injection: Study of 91 cases. Hepatology; 23, 994-1002.*

**To assess the efficacy of ultrasound (US)-guided percutaneous acetic acid (in concentrations of 15%, 20%, 30%, 40%, and 50%) injection for small hepatocellular carcinomas (HCCs) for long-term prognosis, percutaneous acetic acid injection using 15% to 50% acetic acid was performed in 91 patients with one to four HCCs smaller than 3 cm during the past 6.5 years. During the series of treatment sessions for each patient, the same concentration of acetic acid was used. All tumors could be treated successfully with percutaneous acetic acid injection despite the differences in acetic acid concentration used. The number of treatment sessions to treat similar size of tumor was less when the higher concentration of acetic acid was used. No serious complications occurred as a direct sequela to percutaneous acetic acid injection. None of the tumor treated regrew. The 1-, 2-, 3-, 4-, and 5-year survival rates for 91 patients were 95%, 87%, 80%, 63%, and 49%, respectively. The 1-, 2-, 3-, 4-, and 5-year cancer-free survival rates of these patients were 83%, 54%, 50%, 37%, and 29%, respectively. Both liver function and size of tumor affected both survival rate and cancer-free survival rate significantly, but the number of tumors did not. The concentration of acetic acid did not affect the survival rate. Percutaneous acetic acid using 15% to 50% acetic acid will be effective therapy for small HCCs for long-term prognosis. (Hepatology 1996; 23, 994-1002).**

*Keywords:* Hepatocellular carcinoma, alcohol injection, acetic acid injection

### PAPER DISCUSSION

The study by Ohnishi *et al.* [1] recommended that acetic acid is the preferred agent to absolute alcohol because acetic acid has the property of

